# When Spinal Neuromodulation Meets Sensorimotor Rehabilitation: Lessons Learned From Animal Models to Regain Manual Dexterity After a Spinal Cord Injury

**DOI:** 10.3389/fresc.2021.755963

**Published:** 2021-12-07

**Authors:** África Flores, Diego López-Santos, Guillermo García-Alías

**Affiliations:** ^1^Department of Cell Biology, Physiology and Immunology, Institute of Neuroscience, Universitat Autònoma de Barcelona and Centro de Investigación Biomédica en Red sobre Enfermedades Neurodegenerativas (CIBERNED), Bellaterra, Spain; ^2^Institut Guttmann de Neurorehabilitació, Badalona, Spain

**Keywords:** upper limb, rehabilitation, neuromodulation, spinal cord injury, activity-dependent plasticity

## Abstract

Electrical neuromodulation has strongly hit the foundations of spinal cord injury and repair. Clinical and experimental studies have demonstrated the ability to neuromodulate and engage spinal cord circuits to recover volitional motor functions lost after the injury. Although the science and technology behind electrical neuromodulation has attracted much of the attention, it cannot be obviated that electrical stimulation must be applied concomitantly to sensorimotor rehabilitation, and one would be very difficult to understand without the other, as both need to be finely tuned to efficiently execute movements. The present review explores the difficulties faced by experimental and clinical neuroscientists when attempting to neuromodulate and rehabilitate manual dexterity in spinal cord injured subjects. From a translational point of view, we will describe the major rehabilitation interventions employed in animal research to promote recovery of forelimb motor function. On the other hand, we will outline some of the state-of-the-art findings when applying electrical neuromodulation to the spinal cord in animal models and human patients, highlighting how evidences from lumbar stimulation are paving the path to cervical neuromodulation.

## Introduction

A shift in scientific paradigm has recently knocked on the spinal cord community's door. Unprecedented results, obtained in three independent laboratories, have demonstrated that people with chronic paraplegia can recover the ability to voluntarily stand and walk while receiving patterns of electrical stimulation to the lumbar spinal cord (1–3). This intervention, hereafter referred to as spinal neuromodulation, consisted of engaging spinal networks through the targeted delivery of patterned electrical stimulation to enable or facilitate motor performance. Never before has an intervention achieved this success, and a promising new avenue of studies will hopefully refute the until now valid statement that spinal cord injuries are uncurable (4).

None of these achievements would have been possible without the extensive experimental research conducted over the last decades. The accumulated knowledge of spinal cord physiology, locomotor function, and rehabilitation among others, and most recently of spinal stimulation, have established strong bases for quickly and efficiently designing and testing spinal neuromodulation in chronic spinal cord injured patients. This scientific success further evidences the necessary synergy between experimental and clinical studies; results obtained from lampreys, rodents, cats and non-human primates have settled a detailed functional map of the brain and the spinal cord and have made it possible to identify, locate and understand the function and connectivity of the spinal networks recipient of the electrical current (5). These reports together with recently published studies, which will be described in the following sections, represent only the tip of the iceberg of the work which still needs to be done before we can roundly state that spinal cord injuries have found a cure. Indeed, spinal neuromodulation has undoubtedly opened a realistic, efficient, safe, painless intervention, but yet requires a vast amount of work before being universally implemented.

Although neuromodulation has received much of the attention (i.e., identifying the stimulation pattern and its properties, determining the number and location of electrodes, revealing the mode of action, etc.), we cannot ignore the fact that electrical stimulation must be delivered concomitantly to the performance of sensorimotor rehabilitation. Thus, instead of considering the combination of these interventions, spinal neuromodulation can be understood as an extended rehabilitation tool, increasing the spinal circuit's excitability to enable the execution of movement (6). If so, this statement intrinsically introduces a new variable, which can have important effects on the efficiency of the neuromodulatory intervention: what does the rehabilitation consist of? Or which movements should be trained?

Herein we will introduce the major findings obtained and some of the concerns which experimental and clinical neuroscientists face when attempting to neuromodulate and rehabilitate motor function in spinal cord injured patients. For this purpose, we will first describe the gross organization of the spinal cord, highlighting some of the similarities and plausible differences with the lumbar spinal cord which may dictate the feasibility of being electrically neuromodulated. Secondly, we will explain the major rehabilitation interventions employed in animal research to promote recovery of forelimb function and their outcomes. Finally, we will describe some of the state-of-the-art results when applying electrical stimulation to the cervical spinal cord in animal models and human patients.

## Can We Attempt to Recover Manual Dexterity?

The critical role that hands play in humankind and their activities is undeniable, and it is very difficult to conceive of our culture and behavior without their flexibility, dexterity and strength. It is not surprising that people with cervical spinal cord injury (SCI) prioritize recovering hand function over other system functions (7). Following the motor recovery obtained in people with paraplegia, a next reasonable step would be for patients to improve arm and hand sensorimotor function. Can the cervical spinal cord be recipient of electrical stimulation?

The cervical spinal segment presents many similarities to the lumbar spinal cord. The butterfly-shaped gray matter hosts the spinal neuron cell bodies and is centrifugally surrounded by the spinal pathways connecting the brain sensory and motor centers with the spinal cord and sensory ganglia. Despite the anatomical and physiological differences, the mammalian spinal cord is very conserved among species, allowing translational studies between animal models and humans. This is especially important for hand movement recovery. For example, despite the differences in the species circuit organization controlling manual dexterity (8, 9), movement gestures to reach and grasp are analogous between rodents and humans (10).

**Figure 1:**
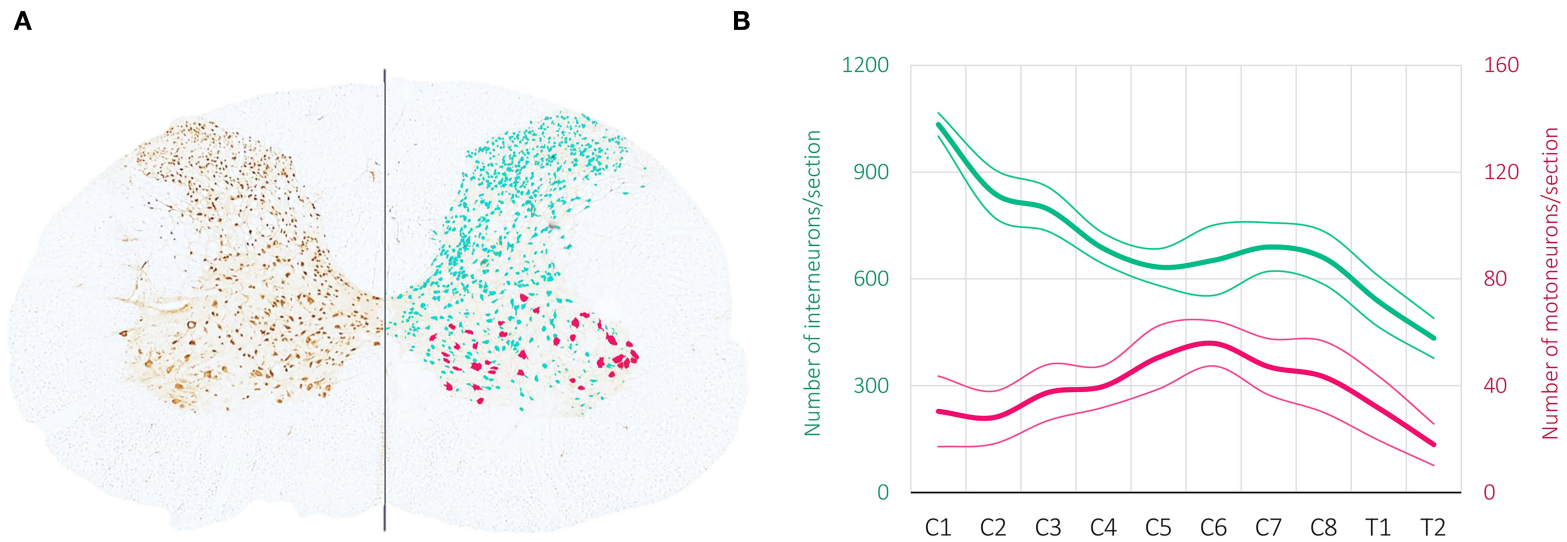
Neurons in the cervical spinal cord. **(A)** Neural marker (NeuN) immunostaining of a transverse section from a rat C6 spinal segment. The left side shows the spinal section raw immunostaining, depicting the neuronal cell bodies distributed along the dorsal, mid, and ventral gray matter. The right side shows the image analysis performed to categorize and subdivide the identified neurons, based on their soma size and location, in interneurons (light green) and motoneurons (red). **(B)** The graph shows the mean ± SE of total interneurons and motoneurons quantified from individual serial sections of the cervical spinal cord from three uninjured adult rats. The number of motoneurons follows the anatomy of the cervical enlargement, with increasing numbers at C5–C7, where the motoneuron pools of the forelimb muscles are located (12). In contrast, the number of interneurons is higher at the most rostral cervical segments and gradually decreases along the rostro-caudal axis.

Unlike locomotion, the neuronal networks controlling skilled hand movement (i.e., reaching and grasping) are still far from being identified, localized and functionally characterized. Albeit far from being completely deciphered, much more is known about the role of the spinal central pattern generators (CPG), the mesencephalic region in the brainstem and the motor cortex in controlling locomotion than in manual dexterity (11). As shown in Figure 1, the number of interneurons is an order of magnitude higher than the number of motoneurons and intuitively suggests the presence of networks that must be tightly related to arm and hand fine motor control. Electrophysiological studies have evidenced the presence of a spinal network at C3·C4, acting as a relay station between the brain and the motoneurons (13). Work from the Isa laboratory has demonstrated the same network in the non-human primate spinal cord and the transitory impairments produced after injury (14) or viral inactivation (15). Although the existence of a cervical CPG for locomotion has been postulated in the cat (16), there is still no evidence demonstrating the existence, location and physiology of a hypothetical cervical CPG for reaching and grasping. Moreover, the newly identified role of the brainstem nucleus medullary reticular formation ventral part (MdV) in executing reaching and grasping movements (17) highlights the new view that endows the brainstem with a strong descending control in hand dexterity (9). Finally, although the motor cortex and the corticospinal tract have been traditionally cataloged as the structures controlling skilled movements (18), studies on rodents (19) and non-human primates (9) have evidenced the maintenance of skilled hand function despite damage to the corticospinal tract and suggest a non-executive but more processing role for this pathway.

Despite their anatomical similarity, the functional differences between the cervical and lumbar spinal cord are obvious. We use our legs to stand, maintain the posture and move, whereas our body biped position allows us to primarily use the arms and hands to transport and manipulate objects. Furthermore, lumbar spinal cord function may rely more robustly on spinal reflexes than the cervical spinal cord, which could be under stronger control from the supraspinal nuclei (20). If we want to neuromodulate an injured cervical spinal cord and replicate the recovery in posture and gait control, it will be necessary to dissect and identify the circuits controlling skilled hand movements which need to be targeted by the electrical stimulation, and we will further need to evaluate the synergies and possible countereffects between the stimulation and the rehabilitation.

Nevertheless, there is a considerable amount of work on upper limb rehabilitation and lumbar neuromodulation which has paved the way for studying the opportunities of neuromodulating the cervical spinal cord (21–24). In the following sections we will describe some of these studies, in animal models and humans, and draw on some of the principles learned if we want that rehabilitation and neuromodulation work together to facilitate skilled hand functional recovery.

## Rehabilitative Training: An Engine for Neuroplasticity

To date, motor rehabilitation is the only therapeutical intervention applied in people with SCI and some of the interventions applied have proven effective in improving patient outcome (25). Other approaches, aiming to repair or regenerate the damaged tissue still have not shown or have failed to prove their potential benefits in human patients as previously reported in experimental animal models (26, 27).

Although physical training has been employed in rehabilitative medicine since the eighteen hundreds (28), optimal training protocols are still not well-established and the underlying neuronal mechanisms resulting in motor improvements remain poorly understood (29). Despite the remarkable benefits of training-based rehabilitation, alone or in combination with other interventions, its systematic application in SCI preclinical studies remains barely settled. Based on the accumulated experience from the clinical rehabilitation centers, animal studies are focused on identifying the mechanisms of recovery, testing the additive synergies with other interventions, and importantly, setting efficient training regimes for achieving consistent functional recovery. However, due to the variety of protocols tested, and the lack of methodological consensus, the optimal parameters still need to be defined (30, 31). Nevertheless, some lessons have been learned, and it is becoming clear that factors such as timing and training intensity, or those limiting training enrolment, have a decisive impact on successful motor recovery and must be carefully considered.

### Defining What, When and How to Train: the Opportunity Window

After an injury to the central nervous system (CNS), rehabilitative training aims to recover sensorimotor function by promoting adaptive neuroplastic changes through repetition of specific movements (32). Similar to what happens during development or learning processes (33, 34), activity-dependent plasticity relies on reshaping the residual neural circuit connectivity, ultimately improving their functionality (35–37). However, neuroplasticity does not always translate into functional improvement; if not applied during particular time windows, or under specific conditions after injury, it may lead to suboptimal or even maladaptive neural changes (38–40). Therefore, it will be crucial to implement rehabilitation protocols according to the specific pathophysiological stage of the injured spinal cord thus enabling activity-based plasticity to make the most of these limited *windows of opportunity*, a concept long used in stroke research (39).

#### The Training Task

**Figure 2:**
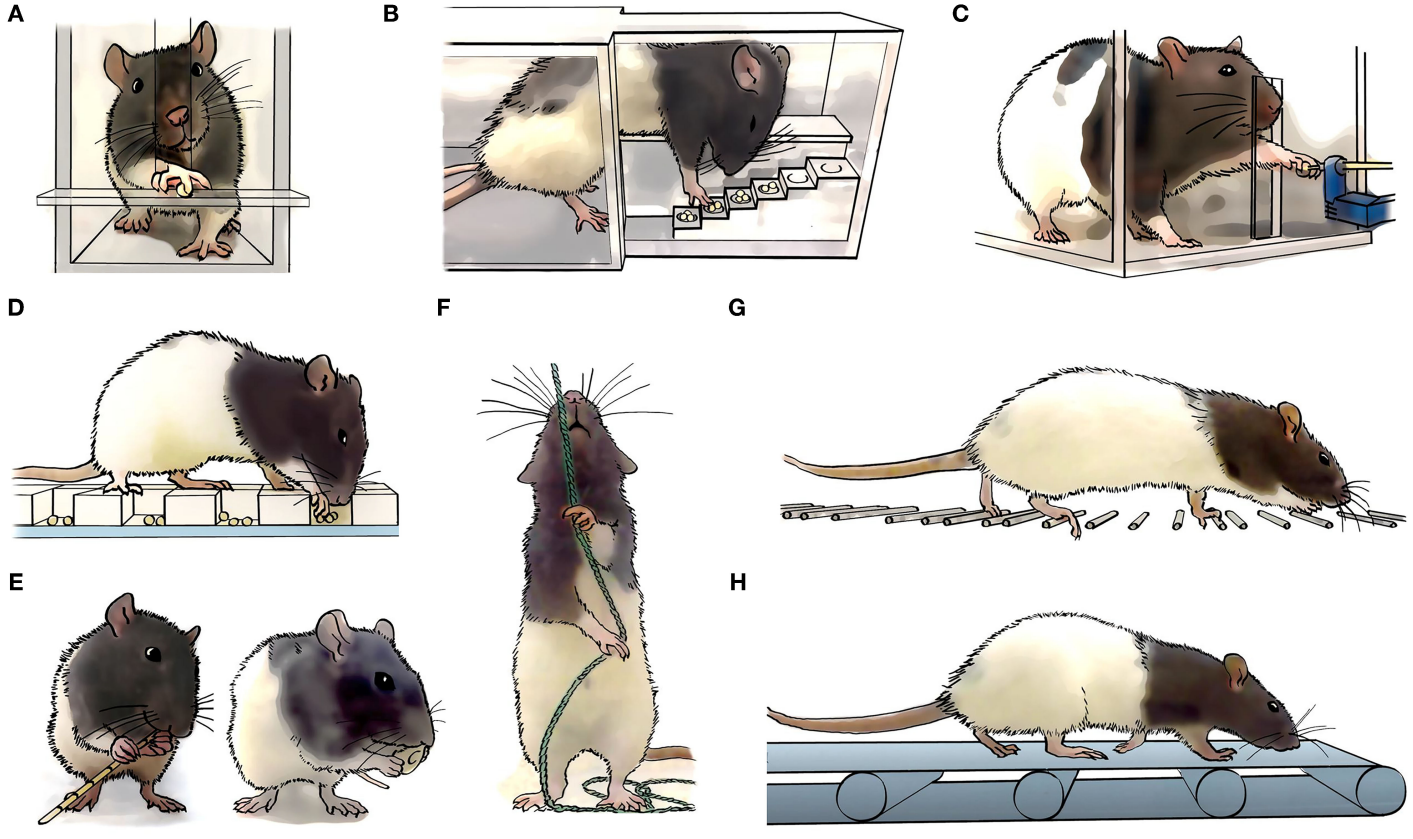
Task specific forelimb motor assessment and rehabilitation. Long-Evans rats are commonly used to study forelimb motor control. In comparison to other rat strains, Long-Evans rats rapidly learn dexterous tasks, which can be associated with a larger cortical motor representation map (41). Different specific motor tasks are being used to assess the animals skills and abilities, including **(A)** single pellet reaching and grasping, **(B)** reaching and grasping in a staircase, **(C)** grip strength, **(D)** reaching and grasping form a grid, **(E)** food manipulation, such as pasta or cereals, **(F)** rope pulling, **(G)** horizontal ladder, and **(H)** treadmill locomotion.

**Figure 3:**
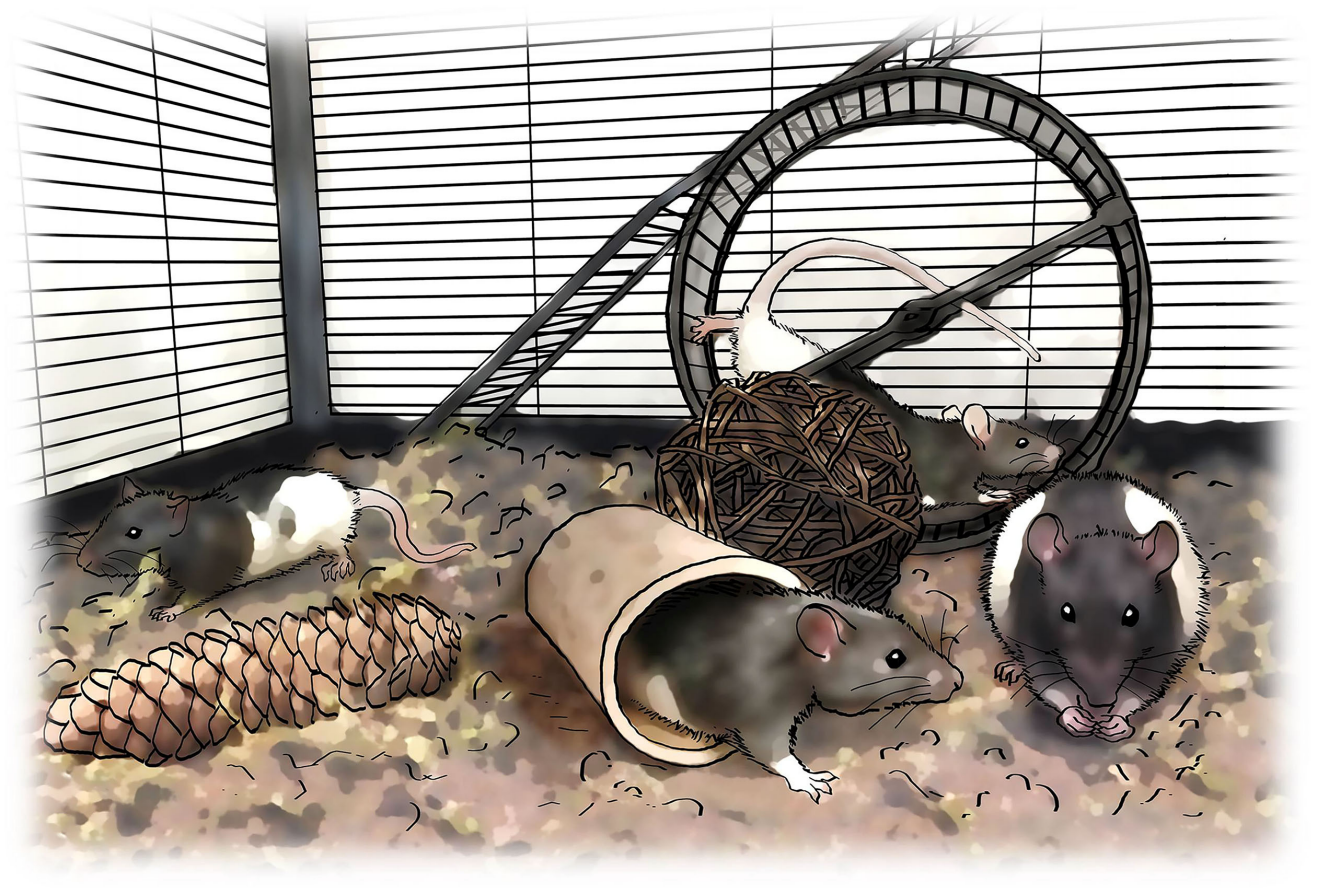
Enriched environment rehabilitation. An alternative to task-specific rehabilitation is to engage the animals in an enriched environment in which they have the chance to voluntarily run along rungs, climb the cage walls, nest with the cage sawdust, manipulate food and run in a running wheel.

The first challenge to face when designing a rehabilitative training program for improving upper limb motor control is deciding which task(s) should be trained. Forelimb motor function is assessed in rodents with cervical SCI through a variety of tests, including over-ground locomotion, horizontal ladder, single pellet retrieval, grip strength, rope pulling and food manipulation [for a detailed review see (30)] (Figure 2). Among them, reaching and grasping-based paradigms, including single-pellet reaching and grasping (SPRG) implemented by Whishaw (42), Montoya staircase pellet retrieval (43), or seed/pellet retrieval from a grid floor (44) are the main methods chosen for rehabilitative training after cervical SCI. Rodent studies show that training a particular movement (i.e., task-specific training) induces recovery mainly in *that* specific trained task, although it may interfere with the performance of untrained tasks (35, 44). For instance, reaching and grasping training improved motor outcome in the same task but interfered with horizontal ladder performance in rats (35, 45, 46). Similarly, locomotor training worsened reaching and grasping scores in rats with unilateral dorsal funiculus section (44). On the other hand, some studies report improvement in non-trained movements (44, 47). For example, horizontal ladder or single-pellet retrieval rehabilitation not only induced recovery in the trained task, but some improvement also transferred reciprocally between tasks, and even to a novel, untrained pellet retrieval task (i.e., the staircase) (47). As the degree of transferability seems fairly unpredictable based on movement similarities, choosing the best rehabilitation task may rely on the severity and type of deficit produced by the injury. Thus, tasks training fine digit control (e.g., SPRG) may result more appropriate for the recovery from mild or moderate injuries, or those affecting distal rather than proximal movements. On the other hand, treadmill locomotion (48) and forced/voluntary running wheel (49), which involve strength/cardiovascular resistance, can also promote recovery of forelimb movements. However, these trainings may entail a confounding factor when interpreting motor improvement as it is difficult to dissect the neural bases from the exercise-induced benefits (31). Additionally, environmental enrichment has been used as a non-task-specific forelimb rehabilitative training after SCI (50–52). This consists of supplementing the animal's home cage with diverse objects, such as ropes, ladders, wheels, cones, bridges or pellet dispensers, that motivate the animal to increase its general motor activity (Figure 3). Although environmental enrichment has been reported to improve forelimb motor performance (50, 52, 53), motor output in a particular non-trained task may be interfered with depending on the tasks included as enrichment, which should be chosen carefully.

#### The Time of Onset

Clinical experience shows that rehabilitation only has a remarkable impact on functional outcome if implemented during the first few months after injury (54, 55). Following this period, which is highly susceptible to plastic changes, motor performance reaches a plateau and further improvements are scarce. Both animal and clinical studies point to the same principle: an early onset (within the first week in animal studies) leads to motor improvement (35, 56), but delaying the start of the training remarkably hinders functional recovery (57–59). Rats substantially recovered forelimb motor function when reaching and grasping training was applied as early as four to seven days after injury (35, 45). In contrast, those improvements were practically absent when a similar rehabilitative regime was initiated 2 months after injury (60), although some authors describe certain improvements when starting at the same timepoint (61). Similarly, macaque monkeys with a corticospinal tract lesion recovered dexterous hand movements during the first 1–2 months if food retrieval training was initiated immediately after injury, but if training onset was delayed 1 month, hand performance remained deficient even after 3 months of training (59). Delayed onset is also associated with increased use of alternative movements (i.e., compensatory strategies) (59, 60). These compensatory strategies probably emerge as a spontaneous form of motor learning during the transient period of enhanced plasticity that occurs immediately after an injury to the CNS (62), setting suboptimal circuitry rearrangements. Compensatory movements can be prevented if task-specific training is introduced on time to appropriately shape this plastic potential, promoting restorative rather than compensatory motor recovery. On the other hand, rehabilitative research after stroke shows that earlier onset is not always better and introducing training too soon after CNS insult leads to deleterious effects (39, 63, 64). It is not clear whether this might also apply to SCI, but it has been observed that if onset of reaching and grasping training is established at 4 days after a cervical SCI, motor performance in a non-trained task (i.e., horizontal ladder) is impaired (35). Notably, this deleterious effect was prevented if reaching training was delayed to 12 days post-injury, without affecting the recovery of the trained task (45).

#### Intensity and Dosage

When designing a rehabilitative protocol, researchers must also define several parameters related to the amount of effort performed by the animal throughout the training: the total number of training sessions, their frequency, the number of gesture repetitions per session and the number of repetitions per time unit (i.e., speed). All these factors strongly influence the effectiveness of rehabilitation, both in animal models (65, 66) and humans (32, 67). In human patients with SCI, it has been estimated that maximal functional recovery requires high intensity training, understood as >60 total training sessions of at least 1.5 h per session, administered daily (32), although severity, type of injury and trained task may modify these predictions. However, it is difficult to extract clear conclusions from animal studies as reporting training intensity and/or dosage details is often omitted (31). Typically, during reaching and grasping rehabilitation, the delivery of 20–40 pellets within a 10-min daily session is enough to observe certain motor improvement (35, 45, 68). Recent studies suggest that there is potential for stronger motor improvement if training is delivered at higher intensity rates (66, 69). In cervical SCI rats, reaching and grasping rehabilitation led to motor recovery when applied early after injury but resulted ineffective if administered in the chronic stages of SCI unless the rehabilitation intensity was tripled (60). Another study showed that *ad libitum* access to an automated device for reaching and grasping rehabilitation allowed injured rats to undergo self-directed training intensity and to naturally segregate the animals as low- and high-performers based on their training strategy (66). Those animals self-engaged in high-intensity training (i.e., higher number of total attempts and performed at higher speed) displayed better motor recovery. However, values over a particular amount and intensity of rehabilitative training did not involve any benefit in recovery, suggesting that there is a limit after which further recovery cannot be achieved (66). Moreover, excessive training intensity may lead to detrimental effects (i.e., repetition-associated musculoskeletal pathologies (70, 71) with no further benefits, highlighting the relevance of establishing the optimal high-dose limit of rehabilitation-induced recovery.

### Rehabilitation Enrolment: Do Not Miss the Chance

Unlike body-support treadmill locomotion, forelimb rehabilitation often involves training tasks that require a high input of voluntary drive. This volitional component becomes particularly evident after SCI, when the animal struggles to execute the task and the relationship between effort and reward becomes unbalanced. As observed in the clinical setting, poor patient enrolment can severely compromise the successful execution of any rehabilitation protocol (72), and even those carefully designed to take maximum profit from the rehabilitative training would turn out to be ineffective. Several strategies aimed to ensure the animal's engagement in training have been explored with favorable results, although some associated drawbacks should also be acknowledged (30, 73).

As most of the training tasks employed to rehabilitate skilled forelimb function are based on food-associated rewards (e.g., single-pellet reaching and grasping or seed retrieval), the animal's motivation can usually be maintained by increasing the hedonic value of the food, or by restricting the amount of available food in their home cage (35, 56, 74). In these cases, highly palatable food should be restrained to isocaloric substitutes to minimize undesired effects on satiation and/or metabolism. If food restriction is applied, the severity of deprivation must be carefully controlled, since hungry animals tend to increase their number of attempts at the expense of worsening their success rate due to higher anxiety-like states (75).

A key aspect in rehabilitation engagement relies on the duration of each training session. Specific forelimb training tasks are typically trained by placing the animal in a particular setting or apparatus for a short (i.e., some minutes to 1–2 h) time. This unavoidably limits the amount of training received per day, but also establishes a fixed time during which animals are trained, usually during the light period of the day. Approaches consisting of free access to training overcome these limitations and allow the animal to train steadily and during the night, which coincides with the active phase of the rodent's circadian cycle. Environmental enrichment is one of these *ad libitum* approaches, but it involves a non-task-specific training as stated above and the amount of training performed by each individual is difficult to monitor (50–52). Recently, diverse automated systems (e.g., automatic pellet dispensers) have been developed so they can be coupled to or integrated in the animal's home cage allowing for free access to forelimb task-specific training (76–78). These studies report that both intact and injured rats self-engage in reaching and grasping training more prominently during the dark (66, 79), and achieve higher amounts of rehabilitative training by self-enrolment than manual training after cervical SCI (66, 76). However, the quantity of training performed by each subject is difficult to control as it relies on the animal's will, leading to high inter-subject variability in training amount and intensity performed as well as its progression throughout the rehabilitation period (66).

It is not surprising that, after lateralized damage to the CNS, some subjects rely on the unaffected forelimb to compensate for the loss of function in the impaired paw. This leads to what is known as *learned nonuse* (80) and exacerbates the impairment of the ipsilesional side since it discourages the use of the impaired forelimb (typically corresponding to the originally preferred limb) and also mobilizes skill learning-associated plastic changes that interfere with functional recovery (39, 81). Several strategies can be employed to prevent the use of the contralateral forelimb after cervical SCI. For instance, Montoya's staircase is designed so the animal can only reach the pellets with a particular forelimb. Whishaw's reaching and grasping task can be adapted to force the use of the affected limb by placing the pellet aligned with the outer margin of the window (31, 79), or by using lateralized windows accomplishing the same function. Forced use of the affected forelimb has also been encouraged by restricting the movement of the unaffected paw with a cast (56, 81). After corticospinal tract injury in rats, this strategy led to improved motor performance on the horizontal ladder and activity-dependent intraspinal reorganization, whereas immobilizing the animal's impaired forelimb impeded functional recovery (81). Nevertheless, it must be considered that modifications in the posture or gesture adopted by the animal (especially quadrupeds) will be affected in a manner that hinders accurate comparison with existing data, particularly electromyographic or kinematic data which could be notably affected.

### Passive Exercises: When Willing Is Not Enough

The interventions described above require long-lasting active voluntary activity. However, it is worth mentioning those interventions that are applied with subjects who are physically very weak and unstable and cannot engage in such demanding tasks. Passive physiotherapy has mainly been studied in humans with paraplegia, and there is very little literature on animal work. Thus, there is scant information available on hindlimb and forelimb function. Passive movement therapies are mainly aimed at promoting plasticity by acting on the sensory drive to produce changes in synaptic efficacy between afferents and alpha motoneurons (34). Sophisticated body-weight supported treadmill training (82, 83), passive cycling (84), functional electrical stimulation (85) or direct strengthening and stretching exercises (86) are employed to exercise the hindlimb. Arm- and hand-function passive exercises are, by contrast, based on the use of assisted robotics alone or in combination with neuromuscular electrical stimulation (87).

Animal studies have shown that passive cycling improves cardiac function (88), reduces spinal hyperreflexia (89) and promotes cortical reorganization (90) in animals with thoracic spinal cord injuries. On the other hand, the results from body-weight supported treadmill training have revealed an astonishing plasticity of the spinal cord, allowing spinalized adult rats and cats to take steps (91, 92) and resolved the bases for the subsequent application in human patients. Unfortunately, thus far, body-weight supported treadmill training has not been as successful as expected for humans to recover locomotion (93), probably due to incompatibilities in translating the technical characteristics of treadmill training to over-ground locomotion (94, 95). Although passive exercise has not been applied after cervical SCI for forelimb control recovery in rodents, future studies where injury severity does not allow for active training (either voluntary or forced), particularly in early post-lesion phases, would benefit from including passive training as part of their therapeutic intervention.

### Plasticity-Promoting Strategies: Broadening the Window

As previously mentioned, a temporary window of heightened plasticity appears after SCI during which physical activity, either spontaneous (i.e., everyday movements) or through rehabilitation, can drive meaningful structural changes leading to functional recovery (96). Many of the efforts in SCI research have been dedicated to enhancing, prolonging or retrieving this neuroplastic potential beyond the subacute stages after the injury. Diverse approaches have been explored to promote axonal growth and collateral sprouting, most of which aim to either overcome the *extrinsic* inhibitory environment around the lesion, or to stimulate the *intrinsic* regenerative capacity of neurons. Although many of these attempts successfully achieved structural reorganization (i.e., axonal growth and higher fiber density due to collateral sprouting) (97), there is growing evidence that training might be essential for neuroplasticity-promoting treatments to endow these anatomical changes with functional meaning, enabling recovery (31, 44, 68, 98).

#### Overcoming the Inhibitory Environment

Glial proliferation and scar formation are relevant extrinsic plasticity inhibitors (99). Rolipram, a selective cAMP phosphodiesterase inhibitor, reduces microglial function and proliferation (100) and attenuates the formation of the glial scar after SCI (101), facilitating a permissive environment for axon growth. Although rolipram improved paw placement and locomotion (101, 102), it was not able to further improve motor recovery when co-administered with daily rehabilitation (50, 53). Particular constituents of the extracellular matrix, including chondroitin sulfate and keratan sulfate proteoglycans, are potent axon growth inhibitory molecules within the glial scar that become upregulated after the injury (103, 104). Digestion of these components with chondroitinase ABC (44) or keratanase II (105) promotes axon regeneration and plasticity after SCI, leading to functional recovery when applied together with task-specific forelimb training in rats. Chondroitinase ABC also proved to generalize motor recovery to untrained tasks even with a delayed rehabilitation onset (i.e., 4 weeks post-injury) (58). Axon guidance molecules such as the Wnt family alter the neuroplastic potential after SCI. Besides orchestrating axon growth and direction during development, Wnts are also reinduced after SCI to regulate axon regeneration/sprouting, repelling descending corticospinal tract axons (106). Inhibiting cortical expression of Ryk, the Wnt receptor that mediates repulsive effects, or administering antibodies against Ryk, resulted in increased corticospinal axon sprouting in the spinal cord and enhanced recovery of reaching and grasping following a cervical SCI when combined with task-specific rehabilitation (107). Similarly, sequential application of Nogo-A [a myelin-associated neurite outgrowth inhibitor) (108)] antibodies and rehabilitative training induced contralateral axon sprouting and improvement in skilled forelimb function after cervical SCI (109). Non-human primates also benefit from Nogo receptor blockade after SCI and show corticospinal sprouting below the injury and improved forelimb use that remained at least 2 months after treatment cessation (110).

#### Promoting the Neuron's Intrinsic Plastic Capacities

Diverse approaches aiming to enhance intrinsic rewiring potential have been combined so far with forelimb rehabilitation. One of these works attempted to promote new connections specifically between the corticospinal tract and the reticulospinal tract in the brainstem to enable a detour for descending signals and hence functional recovery after a cervical dorsolateral quadrant section (98). Thus, viral overexpression of brain-derived neurotrophic factor (BDNF), a promoter of collateral sprouting (111) was induced in cortical motor neurons, whereas the chemoattractant neurotrophin 3 (NT-3) was overexpressed in reticular neurons, encouraging CST collaterals to grow toward them (98). This approach promoted forelimb functional recovery only when combined with skilled reaching training, resulting in task-specific improvements. Interestingly, these effects were independent of collateral sprouting of the CST or RtST and remain to be further explored. Dietary supplementation with the omega-3 fatty acid docosahexaenoic acid (DHA) also resulted in functional recovery following pyramidotomy and cervical SCI (112), particularly when combined with forelimb training (113). DHA induced sprouting of CST and serotonergic fibers into the denervated side below the lesion (113), possibly by increasing BDNF levels, among other mechanisms (114, 115). Stimulation of BDNF release, together with potentiation of serotonergic activity within the spinal cord, also takes place after exposure to acute intermittent hypoxia (AIH), a plasticity-promoting approach consisting of brief exposures to reduced oxygen levels alternating with normo-oxygen breaths (116–118). AIH has proved to reopen a period of enhanced plasticity when applied at four (116, 117) or even at 8 weeks (118) post-injury, leading to task-specific functional recovery exclusively when combined with forelimb training. Similarly, reintroducing inflammation through systemic injection of lipopolysaccharide re-established a heightened-plasticity state at 8 weeks post-injury, allowing reaching and grasping training to recover its efficacy to induce CST sprouting into the spinal gray matter and skilled functional improvement (60). A recent work reports that combining task-specific rehabilitative training with inhibition of PTEN (phosphatase and tensin homolog), an intrinsic negative regulator of axon regeneration (119), promotes CST regeneration beyond the lesion site and recovery of reaching and grasping after cervical SCI (120). The onset time for this intervention was also delayed (i.e., 4 weeks) after injury, further supporting that time-dependent plasticity decay after injury can be successfully counteracted through several pharmacological manipulations to prolong activity-driven recovery.

## Spinal Neuromodulation: An Added Tool for Facilitating Rehabilitation

The engagement of spinal circuitries, by delivering electrical stimulation patterns, facilitates the performance of the rehabilitated movements. The International Neuromodulation Society defines neuromodulation as “the alteration of nerve activity through targeted delivery of a stimulus, such as electrical stimulation or chemical agents, to specific neurological sites in the body.” Here, we will refer to neuromodulation as the modulation induced by electrical stimulation. Spinal neuromodulation was originally used to mitigate chronic pain (121, 122). Studies with multiple sclerosis patients, who received epidural spinal cord stimulation to relieve uncontrollable pain, also showed gains in voluntary motor control (123). Remarkably, once the stimulation stopped, the improved function did not revert, suggesting that electrical stimulation was producing some sort of change in the nervous system.

### The First Steps: Learning From Lumbar Neuromodulation

Most of the studies on neuromodulation have been performed on the lumbar spinal cord aiming to gain hindlimb motor control. This is probably due to the longer tradition of studying locomotion.

**Figure 4:**
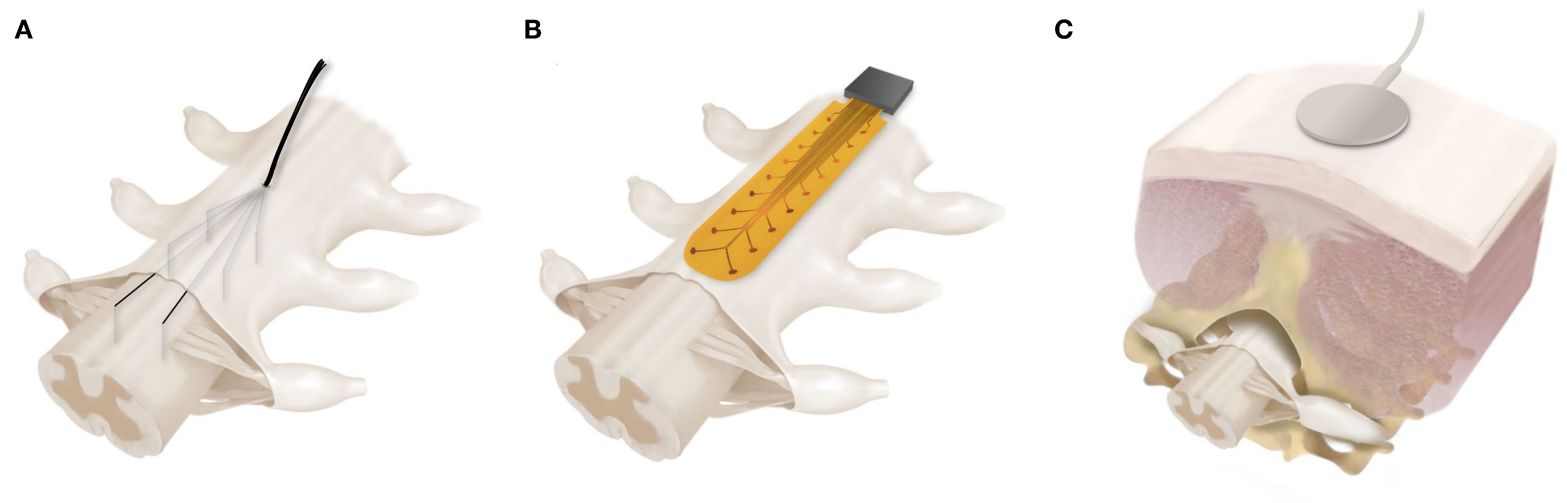
Spinal cord electrical stimulation. Different approaches have been developed in the last years to neuromodulate the spinal cord. **(A)** Intraspinal electrodes within the spinal gray matter, close to the motoneuron pools; **(B)** Epidural electrode arrays are placed over the dorsal side of the spinal cord fixed to the outer side of the meningeal layer; **(C)** Transcutaneous stimulation is delivered by big size adhesive electrodes which are placed percutaneously on the back skin.

The delivery of electrical stimulation has been evolving over the years, from a very invasive approach using intraspinal electrodes implanted in the spinal parenchyma, to single or arrays of electrodes sutured to the epidural layer surrounding the dorsal surface of the spinal cord, and most recently to transcutaneous stimulation, which delivers the current through adhesive electrodes placed on paravertebral or midline skin (Figure 4).

Intraspinal electrodes implanted in the spinal ventral horns directly activate close spinal motoneurons and can robustly evoke complex hindlimb movements in frogs [reviewed in (124)], cats (125–127) and rats (128, 129). By adapting externally controlled machine learning algorithms, the electrical current was delivered through a combination of selective electrodes within an array that enabled decerebrated cats to generate flexor and extensor movements of the hindlimbs, producing bilateral weight-bearing stepping (130). The invasiveness of the procedure makes it less attractive than more recently developed techniques; however, efforts have focused on translating this approach to humans by studying its functionality and mechanical stability in bigger mammals (131).

Epidural electrodes offer a less invasive approach. Although surgery is required to expose the spinal cord and fix the single or electrode arrays on the meninges layer covering the spinal cord, there is no need to penetrate the spinal parenchyma, thus avoiding tissue damage. However, some difficulties may arise due to electrode migration, inflammation or electrode failure (132). Depending on the stimulation intensity, epidural stimulation can evoke early, middle and late reflex latency responses in the hindlimb muscles, which correspond to direct motor, monosynaptic and polysynaptic activation, respectively (133). Therefore, compared to intraspinal stimulation, an additional advantage of epidural stimulation is the nature of the neurons activated.

Using a pair of implanted wire electrodes on the lumbar spinal cord, stepping was evoked in decerebrated cats (134), spinalized cats (135, 136), and spinalized rats (137) while the animals were placed on a treadmill. Electrical stimulation can be combined with pharmacological modulation; injection or delivery of serotoninergic agonist drugs showed additive effects, leading to better stepping kinematics, in rats with complete (138, 139) or incomplete (140) spinal cord injuries. Importantly, the stimulation parameters have been carefully identified, as well as the optimal placement of the electrodes, which have shown to be crucial for enabling proper steps in spinalized rats, whose kinematic and muscle recruitment resembled those of uninjured rats (141). The development of soft multi-electrode arrays which topographically extend over the dorsum of several spinal segments (142) allowed the specific stimulation under real-time processing of gait kinematics and locomotor performance to optimally readjust the hindlimb kinematic for stepping (143).

From the clinical studies, it is mandatory to mention the work done by the group of Harkema and colleagues. Chronic complete SCI (AIS A-B) patients regained voluntary control of leg movements while receiving epidural lumbosacral stimulation together with extensive rehabilitation protocols (144), but also immediately after electrode implantation (145). Subsequent studies have shown that following an intervention period, motor recovery included standing (146) and stepping (3) recovery, even in the absence of stimulation.

The most recently developed approach is the use of transcutaneous electrical spinal stimulation. Without the need for surgery, adhesive stimulating cathode electrodes are placed at single (147) or multiple sites (148) along the back, and the anode electrode on the hips. Transcutaneous stimulation is generally delivered using the “Russian current” method, in which a carrier frequency of 2.5–10 kHz alternating current is applied in 50 Hz rectangular bursts (149) and is painless (148). However, carrier frequency stimulation appears better tolerated than conventional stimulation by neurologically intact participants only at low intensities, whereas both stimulation protocols are indistinguishable once the threshold to evoke spinal motor potentials is reached (150). Nevertheless, Kumru et al. have shown that subthreshold stimulation influences the spinal circuitry more efficiently than higher stimulation intensities (151), reinforcing the use of painless transcutaneous stimulation as a tool to modulate the spinal cord. A recent meta-analysis, including a total sample of 55 persons with SCI showed that transcutaneous electrical spinal stimulation induced muscle activation in the lower and upper limbs. The studies reported an increase in motor response measured by recording surface electromyography, voluntary movement, muscle strength, or function (152).

However, transcutaneous stimulation is scarcely studied in animals. Unstable fixation of the adhesive electrode on the animal's skin and the difficulty placing the electrodes identically during longitudinal studies limit its implementation and have led to the development of transvertebral electrical stimulation, in which electrodes are implanted into the vertebral spinous processes, with a mode of action and muscle responses analogous to those evoked by transcutaneous stimulation (153).

### Cervical Neuromodulation to Regain Manual Dexterity

There are fewer neuromodulation studies conducted on the cervical than on the lumbar spinal cord, and this is probably attributable to the higher complexity of controlling discrete goal-oriented movements with the hands than of rhythmic stepping with the legs. However, as described below, all studies are showing a parallel trend as that for the lumbar spinal cord, with a similar mode of action, suggesting that the beneficial effects of lumbar neuromodulation can be replicated in or interpolated to the cervical spinal cord (Table 1).

**Table 1:** Summary of the most relevant animal studies on cervical spinal neuromodulation.

		**Injury**		**Spinal stimulation**		**Functional assessments**		**Long-term intervention**			
**Reference**	**Species**	**Type**	**Level**	**Type**	**Level**	**Electrophysiology**	**Behavior**	**Regime**	**Stim. parameters**	**Training**	**Major findings**
Moritz et al. (154)	Macaque monkey	N/A	N/A	Intraspinal	C6-T1	Mapping of spinally-evoked motor responses (SEMR) and forelimb movements (by pulse trains).	None (anesthetized)	N/A	N/A	N/A	Arm/hand movements (flexor predominantly) evoked at most of stimulated sites. Coactivation of two to six muscles found at half of sites. Responses elicited from dorsal and ventral horn and from fiber tracts.
Zimmermann et al. (155)	Macaque monkey	N/A	N/A	Intraspinal	C6-T1	Mapping of SEMR and forelimb movements (by pulse trains).	None (anesthetized)	N/A	N/A	N/A	Coordinated functional arm/hand movements evoked by long trains at one stimulation site. R&G movement required stimulation of only two spinal sites.
Sharpe and Jackson (156)	Macaque monkey	N/A	N/A	Intraspinal, subdural, epidural	C5-C7	Dorsoventral mapping of SEMR and forelimb movements (by single- or train-pulses); paired subdural-intraspinal stimulation.	None (anesthetized)	N/A	N/A	N/A	Motor effects of ventral stimulation mainly mediated by direct activation of motoneurons. Dorsal stimulation increased trans-synaptic excitation mediated by descending projections, afferent inputs and/or local interneurons. Subdural stimulation was more specific than epidural or intraspinal.
Sharma and Shah (157)	Rat	N/A	N/A	Epidural	C6 and C8	SEMR (by single- and paired-pulses, at multiple frequencies) at rest, during volitional motor task, and under anesthesia.	SPRG	N/A	N/A	N/A	SEMR with three different waveforms—early, middle and late-, corresponding, respectively, to activation of motoneurons directly, type-I sensory afferents and wider spinal interneuronal circuits. Middle and late responses, but not early, modulated by repeated stimulation protocols and volitional motor activity.
Greiner et al. (158)	Macaque monkey	N/A	N/A	Epidural	C3/C4 and T1/T2	Mapping of SEMR (by single- and train-pulses) through medial and lateral electrodes under anesthesia; continuous stimulation (50 Hz) during volitional motor task.	Reaching, grasping and pulling	N/A	N/A	N/A	Stimulation of individual roots achieved with lateral (better than medial) electrodes. Motoneuron recruitment trans-synaptically *via* direct excitation of sensory afferents. Modulatory effect of stimulation was movement-phase-dependent.
Guiho et al. (159)	Macaque monkey	N/A	N/A	Epidural	C7	SEMR (by single- and train-pulses) through surrounding multielectrode cuff; paired ICMS-epidural SCS	None (anesthetized)	N/A	N/A	N/A	Ventral stimulation elicited robust forelimb movements even at low intensities and high frequencies. Dorsal stimulation facilitated supraspinal-evoked responses, especially at intermediate stimulation frequencies.
Guiho et al. (159)	Macaque monkey	N/A	N/A	Transcutaneous	C3/C4 and T1/T2	Paired ICMS-transcutaneous SCS (“Russian current”).	None (anesthetized)	N/A	N/A	N/A	Transcutaneous stimulation effective (less than epidural) at facilitating supraspinal-evoked responses, especially at intermediate stimulation frequencies.
Sunshine et al. (160)	Rat	Lateralized contusion	C4-C5	Intraspinal	C3-T1	Mapping of SEMR and forelimb movements (by pulse trains).	None (anesthetized)	N/A	N/A	N/A	Motor thresholds and number of movement-evoking sites unchanged by SCI. Three and 6 weeks after injury: extensor-predominant movements and restricted muscle synergies. Nine weeks after injury: recovery of full robust arm/hand movements.
Zimmermann and Jackson (161)	Macaque monkey	Reversible inactivation (muscimol)	Hand region of M1 (cortex)	Intraspinal (closed-loop)	C4-T1	SEMR (by pulse trains) at rest; closed loop system: biphasic pulses delivered 100–200 ms after M1 neuron spiking during volitional motor task.	Reaching, grasping and pulling	N/A	N/A	N/A	During closed-loop stimulation, animals with disrupted corticospinal control displayed better EMG, movement amplitude and grasp-pull success than when the stimulation was off.
Alam et al. (162)	Rat	Dorsal funiculi crush	C4	Epidural	C6 and C8	SEMR (by single-pulse) at diverse electrode configurations, at rest; continuous stimulation (40 Hz) during volitional motor task.	Grip strength	N/A	N/A	N/A	SEMR were evoked in all muscles also after SCI. Simultaneous C6 and C8 stimulation produced better muscle recruitment and higher grip strengths than stimulation at one site.
Samejima et al. (163)	Rat	Lateralized contusion	C4	Epidural (brain-computer-spinal interface)	C6	Pre/post-injury cortical decoding for forelimb movement; spinal RMT (by pulse trains) at rest. BCI: biphasic train pulses (50–100 Hz) delivered after sensorimotor cortex local field potentials during volitional motor task.	Lever-pressing task	N/A	N/A	N/A	Intracortical local field potentials were stable markers of forelimb movement intention before and after SCI. Forelimb function improved after injury when brain-controlled epidural stimulation was on.
Kasten et al. (164)	Rat	Lateralized contusion	C4-C5	Intraspinal	C6-T1	Spinal stimulation resting motor thresholds (RMT)	SPRG, forelimb asymmetry	ISMS: 7 h/day, 5 d/week, 12 weeks; start 4 weeks after injury	Continuous biphasic pulses (at RMT), 4 ± 1.5 Hz	SPRG after each ISMS session	Injured animals performed better in SPRG when stimulation was given before reaching and grasping, possibly priming the system for movement execution.
McPherson et al. (165)	Rat	Lateralized contusion	C4-C5	Intraspinal (closed-loop)	C6-C8	Spinal stimulation RMT (by single-pulses)	SPRG	ISMS: 5–8 h/day, 5 d/week, 13 weeks, start 6 weeks after injury	Biphasic pulses (at 90% RMT), delivered 0.2 ms after EMG activity (closed-loop) or at EMG-independent pattern (open-loop)	SPRG (30 min/day) during ISMS	Injured rats receiving closed-loop ISMS plus rehabilitation showed better SPRG performance than open-loop ISMS+rehabilitation or only-rehabilitation rats. Therapeutic gains remained for three additional weeks without stimulation.
Alam et al. (166)	Rat	Dorsal funiculi crush	C4	Epidural	C6 and C8	Spinal stimulation RMT (by train pulses).	SPRG	Intense functional assessment: 3 d/week SEMR threshold + 3 d/week SPRG+stim. 10 weeks, start 1 week after injury	Monophasic pulses (60–70% RMT), at 20, 40 and 60 Hz.	SPRG (20 min/day) during on/off stimulation	Injured rats improved SPRG performance during bipolar C6–C8 stimulation compared to monopolar stimulation or no stimulation. C6–C8 stimulation recovered pre-injury-like muscle synergies.
Rascoe et al. (167)	Rat	Complete hemisection	C4	Epidural (closed-loop)	C6 and C9	Spinal stimulation RMT (by train pulses).	SPRG, horizontal ladder, treadmill, grooming and rearing	Epidural SCS during unsupervised overnight activity: 7 h/session, 6 d/week, 12 weeks	Biphasic pulses (at 90% RMT), delivered after EMG activity onset, single or at 500 ms, 40 Hz trains.	Forelimb testing (1 d/week)	Proof of concept for long-term implementation of EMG-triggered closed-loop epidural stimulation (effects on skilled forelimb function not analyzed).
Song et al. (168)	Rat	Unilateral section	Pyramids	Transcutaneous (plus cortical stimulation)	C4-T2	MEP facilitation by spinal-cortical paired stimulation at diverse ISIs, spinal and cortical stimulation RMT.	Horizontal ladder (1–4 w post-stimulation)	tDCS plus cortical stimulation: 27 min/d, 10 days, start 1 week after injury	tDCS: continous current at 1.5 mA	N/A	In intact rats, cathodal tsDCS combined with cortical neuromodulation facilitated MEPs and increased M1 activity/forelimb EMG correlation during locomotion. Daily cortical+spinal neuromodulation after injury restored horizontal ladder performance and CST sprouting.
Zareen et al. (169)	Rat	Midline contusion	C4	Transcutaneous (plus cortical stimulation)	C4-T2	Spinal and cortical stimulation RMT separately.	Horizontal ladder, cereal manipulation (IBB) (1–3 w post-stimulation)	tDCS plus cortical stimulation: 30 min/d, 10 days, start 1 week after injury	tDCS: continous current at 1.5 mA	N/A	Combined cortical and spinal neuromodulation after SCI improved motor recovery and enhanced CST sprouting below and above the injury.
Yang et al. (170)	Rat	Midline contusion	C4	Transcutaneous (plus cortical stimulation)	C4-T2	Spinal and cortical stimulation RMT separately.	Horizontal ladder, cereal manipulation (IBB) (1–4 w post-stimulation)	tDCS plus cortical stimulation: 30 min/d, 10 days, start 11 days after injury	tDCS: continous current at 1.5 mA	N/A	Replication study (169) in an independent lab. Combined cortical and spinal neuromodulation after SCI improved forelimb performance and enhanced CST sprouting.
Sharif et al. (171)	Rat	Midline contusion	C4	Transcutaneous (plus cortical stimulation)	C4-T2	Spinal and cortical stimulation RMT separately.	Horizontal ladder (2–8 w post-stimulation)	tDCS plus cortical stimulation: 30 min/d, 10 days, start 2 w after injury	tDCS: continous current at 1.5 mA	Horizontal ladder: 5 days/week for 6 weeks after stimulation period	Combined cortical and spinal neuromodulation plus rehabilitation enhanced recovery of horizontal ladder performance and CST sprouting compared to rehabilitation only.

*SCS, spinal cord stimulation; SEMR, spinally-evoked motor responses; ICMS, intracortical microstimulation; SPRG, single-pellet reaching and grasping; ISMS, intraspinal microstimulation; tDCS, transcraneal direct current stimulation*.

Intraspinal electrodes implanted in the cervical ventral horns can elicit complex forelimb movements (e.g., reaching and grasping) by the coactivation of multiple muscle activity, as shown in intact anesthetized macaques (154, 155) and uninjured and contused anesthetized rats (160). Awake macaques with muscimol-silenced motor cortex had better electromyographic activity, movement amplitude and grasp-pull success when receiving intraspinal stimulation (161). Moreover, intraspinal stimulation has shown to induce plastic changes in the spinal cord circuits: contused rats had better reaching and grasping performance when stimulation was delivered before the beginning of the testing sessions, priming the system for movement execution (164). In a later study, contused spinal cord-injured rats were rehabilitated for reaching and grasping while receiving intraspinal stimulation, and they not only performed better than non-stimulated rats, but their gains also persisted for 3 weeks without any additional intervention, indicating that intraspinal stimulation has long lasting effects that extend beyond the stimulation period (165). Importantly, motor recovery was only observed under a closed-loop (but not open-loop) procedure in which spinal stimulation was triggered after muscle EMG activity detection, denoting the relevance of temporal tuning of stimulation delivery.

Epidural stimulation also improves reaching and grasping performance in rats with cervical SCI (166). The animal's success improved when the epidural stimulation was applied concomitantly to or before the beginning of the testing. Bipolar stimulation between electrodes implanted in the caudal cervical spinal segments produced the higher reaching and grasping success rates and argue that muscle synergies, which had been impaired with the SCI, returned to pre-injury levels. Recent studies have further investigated in-depth the mechanisms by which epidural stimulation modifies the spinal circuit physiology. Using sophisticated computational simulations together with data obtained from cervical epidural stimulated macaques, it was evidenced that dorsally placed epidural electrodes predominantly recruit spinal motoneurons trans-synaptically through depolarization of sensory afferent fibers (158). Epidural stimulation also recruited descending and ascending fibers (including corticospinal and spinocerebellar tracts and dorsal columns), depending on medio-lateral electrode placement. Indeed, only low-amplitude stimulation at laterally-placed electrodes was able to preserve segmental specificity (i.e., selective recruitment of individual roots). Notably, primary afferent stimulation of upper limb muscles enhanced the motor activity of synergistic muscles only when delivered during a voluntary task (i.e., reaching and grasping), suggesting that the modulatory effect of epidural stimulation is movement-dependent and likely promotes muscle synergies (158). Using a new multielectrode cuff, which surrounds the perimeter of the spinal cord, it has also been possible to selectively stimulate the dorsal and the ventral aspects of the spinal cord in anesthetized macaques (159). As hypothesized, dorsal epidural stimulation trans-synaptically activated the cervical motoneurons, whereas ventral epidural stimulation acted on them directly. Along these same lines, Sharma and Shah (157) explored several stimulation protocols in both anesthetized and awake rats and identified diverse responses recorded in the forelimb muscles. Their results replicate the previously described responses evoked by lumbar spinal stimulation in hindlimb muscles (133) and demonstrate the stimulation's equal mode of action on the cervical and the lumbar spinal cord. These experiments also suggest that not only sensory fibers are susceptible to electrical stimulation, but also spinal neurons can be activated. At increasing stimulation intensities, motor responses with different latencies were recorded and identified as early, middle and late responses. Whereas, early responses probably arose from direct activation of motor efferents, middle and late responses were presumably elicited by activation of type-I sensory afferents and of interneuronal circuitries, respectively. These results evidence that neurons at different locations within the dorsal-ventral axis respond to stimulation applied on the dorsum of the cord and suggest that not only thick sensory fibers but also spinal neurons can be modulated by electrical stimulation.

The first published study on neuromodulation of the cervical spinal cord to improve the recovery of upper limb function in human patients used epidural electrodes (172). Patients suffering from a chronic AIS B cervical SCI (at C5/C6 level) showed better hand control and strength when receiving neuromodulation within the same session and improved in both conditions (with and without stimulation) during the 8 weeks of intervention. At present, most clinical studies focus on testing the effects of transcutaneous stimulation. In a clinical case study, Inanici et al. (173) tested a patient with C3, incomplete, chronic SCI (AIS D) who received rehabilitation phases alternating with and without stimulation. The patient improved sensory and motor function, even when tested without stimulation, and the improvements remained for at least 3 months after finishing the treatment. In another study, hand grip strength was measured in chronic AIS B-C patients (174). Patient hand grip strength was greater when receiving the stimulation and at the end of the 4-week intervention, indicating a physiological improvement and not exclusively restricted to the acute stimulation period. Long-lasting effects were also reported in patients with chronic AIS B, who received transcutaneous stimulation together with the administration of the serotoninergic agonist buspirone (175).

To our knowledge, except for Guiho et al. (159), who studied forelimb motor responses in anesthetized non-human primates elicited by transcutaneous SCS and epidural stimulation, there are no reported animal studies using cervical transvertebral electrical stimulation. However, in a different paradigm, work from John Martin's laboratory explores the effects of combined cortical epidural motor cortex stimulation and cathodal transcutaneous cervical direct current stimulation on motor function recovery (168–171). Notably, when this combined cortical and spinal neuromodulaton approach was applied together with horizontal ladder rehabilitation, forelimb motor improvement and corticospinal sprouting was more evident than in animals receiving rehabilitation only (171).

In summary, all the aforementioned studies evidence that any of the neuromodulatory approaches when applied to the cervical spinal cord enable to some extent voluntary control of previously paralyzed upper limb muscles. A detailed description of the mechanism of actions governing the underlying plasticity remain unknown. However, there is a consensus that the stimulation effects are based on recruiting sensory inputs from the dorsal cord lying under the stimulating electrodes, followed by polysynaptic activation of the spinal neuronal circuits (176). No secondary effects (such as pain or spasticity) have been reported (173, 177, 178). Future directions pursue to develop neuromodulatory “closed-loop” systems and brain-computer-spinal interfaces (163, 167, 179–181), where the stimulation is directly controlled by endogenous biological signals that highly correlate with intentionality, instead of being applied by the experimenter/therapist.

## Conclusions

As recently reported by Morse et al. (4), a recent proceeding hosted by the National Institutes of Health (NIH) aimed to present and discuss the progress, opportunities and priorities for the next decade of spinal cord research clearly expressed the general interest and optimism among scientist, clinicians, patients and general public regarding the potential of spinal neuromodulation to improve motor and other systemic physiological functions in people with chronic spinal cord injuries. It also highlighted the necessity to understand the rehabilitation dose necessary for clinically meaningful effects and to optimize the stimulation parameters to neuromodulate spinal networks at different stages after injury. To fill in these gaps, there is no doubt that animal studies will bring valuable information on the structure and physiology of the cervical spinal cord networks, together with the plastic processes occurring during and after activity-dependent interventions. We need to carefully look back and take advantage of what has been learned from the lumbar spinal cord and draw up a new set up to engage cervical spinal networks to regain the control of skilled arm and hand movements.

## Author Contributions

GG-A, ÁF, and DL-S wrote and edited the review. DL-S and ÁF performed the artwork. All authors contributed to the article and approved the submitted version.

## Funding

This work was supported by Ministerio de Economía y Competitividad (SAF2016-79279-R) and La Marató de TV3 (no. 201713-30) to GG-A.

## Conflict of Interest

The authors declare that the research was conducted in the absence of any commercial or financial relationships that could be construed as a potential conflict of interest.

## Publisher's Note

All claims expressed in this article are solely those of the authors and do not necessarily represent those of their affiliated organizations, or those of the publisher, the editors and the reviewers. Any product that may be evaluated in this article, or claim that may be made by its manufacturer, is not guaranteed or endorsed by the publisher.
